# Craniopharyngioma resection by endoscopic endonasal approach versus transcranial approach: A systematic review and meta-analysis of comparative studies

**DOI:** 10.3389/fonc.2022.1058329

**Published:** 2022-11-30

**Authors:** Min Kyun Na, Bohyoung Jang, Kyu-Sun Choi, Tae Ho Lim, Wonhee Kim, Youngsuk Cho, Hyun-Goo Shin, Chiwon Ahn, Jae Guk Kim, Juncheol Lee, Sae Min Kwon, Heekyung Lee

**Affiliations:** ^1^ Department of Neurosurgery, College of Medicine, Hanyang University, Seoul, South Korea; ^2^ Department of Preventive Medicine, College of Korean Medicine, Kyung Hee University, Seoul, South Korea; ^3^ Department of Emergency Medicine, College of Medicine, Hanyang University, Seoul, South Korea; ^4^ Department of Emergency Medicine, College of Medicine, Hallym University, Chuncheon, South Korea; ^5^ Department of Emergency Medicine, College of Medicine, Chung-Ang University, Seoul, South Korea; ^6^ Department of Neurosurgery, Dongsan Medical Center, Keimyung University School of Medicine, Daegu, South Korea

**Keywords:** craniopharyngioma, endoscopic endonasal approach, transcranial approach, metaanalysis, systematic review

## Abstract

**Introduction:**

The transcranial approach (TCA) has historically been used to remove craniopharyngiomas. Although the extended endoscopic endonasal approach (EEA) to these tumors has been more commonly accepted in the recent two decades, there is debate over whether this approach leads to better outcomes. The goal of this systematic review and meta-analysis was to more comprehensively understand the benefits and limitations of these two approaches in craniopharyngioma resection based on comparative studies.

**Methods:**

We conducted a systematic literature search in accordance with the Preferred Reporting Items for Systematic reviews and Meta-Analyses recommendations using MEDLINE, EMBASE, and the Cochrane Library. A total of 448 articles were screened. Data were extracted and analyzed using proportional meta-analysis. Eight comparative studies satisfied the inclusion criteria. The extent of resection, visual outcomes, and postoperative complications such as endocrine dysfunction and cerebrospinal fluid (CSF) leakage were compared.

**Results and discussion:**

Eight studies, involving 376 patients, were included. Resection by EEA led to a greater rate of gross total resection (GTR) (odds ratio [OR], 2.42; p = 0.02; seven studies) with an incidence of 61.3% vs. 50.5% and a higher likelihood of visual improvement (OR, 3.22; p < 0.0001; six studies). However, TCA resulted in a higher likelihood of visual deterioration (OR, 3.68; p = 0.002; seven studies), and was related, though not significantly, to panhypopituitarism (OR, 1.39; p = 0.34; eight studies) and diabetes insipidus (OR, 1.14; p = 0.58; seven studies). Although TCA showed significantly lower likelihoods of CSF leakage (OR, 0.26; 95% confidence interval [CI], 0.10–0.71; p = 0.008; eight studies) compared to EEA, there was no significant difference in meningitis (OR, 0.92; 95% CI, 0.20–4.25; p = 0.91; six studies) between the two approaches. When both approaches can completely resect the tumor, EEA outperforms TCA in terms of GTR rate and visual outcomes, with favorable results in complications other than CSF leakage, such as panhypopituitarism and diabetes insipidus. Although knowledge of and competence in traditional microsurgery and endoscopic surgery are essential in surgical decision-making for craniopharyngioma treatment, when both approaches are feasible, EEA is associated with favorable surgical outcomes.

**Systematic review registration:**

http://www.crd.york.ac.uk/PROSPERO/, identifier CRD42021234801.

## 1 Introduction

Craniopharyngiomas are calcified embryonic tumors originating from the pituitary gland’s anterior lobe, from epithelial remnants of squamous cell rests of Rathke’s pouch (1, 2). Although craniopharyngiomas are histologically benign (World Health Organization grade I), their complete resection without neurological injury is challenging due to the tumor location (suprasellar, often superiorly extending into the third ventricle) and their relation to critical neurovascular structures, such as the pituitary gland, hypothalamus, infundibulum, ophthalmological systems, internal carotid artery and its branches, anterior cerebral artery-anterior communicating artery complex, basilar artery and its branches, and brain stem (3, 4). Symptoms are often related to surrounding structural compression or infiltration and may include visual disturbance, especially bitemporal hemianopsia, endocrine dysfunction, headache, and hydrocephalus (5).

The primary aims of treatment include tumor elimination; functional outcomes, such as visual, pituitary, and hypothalamic functions; favorable cognitive outcome; and quality of life. Traditionally, the transcranial approach (TCA) has been used to successfully remove these craniopharyngiomas. TCA procedures include the classical craniotomies such as pterional, orbitozygomatic, bifrontal interhemispheric, unilateral subfrontal, and supraorbital approaches (6). However, these approaches have a higher risk of visual impairment, stroke, and other neurologic complications from brain retraction and neurovascular structure manipulation (7, 8). Recently, it was shown that removing craniopharyngiomas in the retrochiasmatic space that extended superiorly into the third ventricle could be accomplished successfully using the purely extended endoscopic endonasal approach (EEA) through the transplanum transtuberculum corridor. Through a subchiasmatic corridor, this approach provides a wide surgical view and allows direct access to tumors without brain retraction and neurovascular structure manipulation (4). However, TCA has surgical advantages over EEA since it avoids damage to the nasal canal or traversing a contaminated field and provides a larger view of lateral tumor extension. The European Association of Neurosurgical Societies recommended the use of TCA for craniopharyngiomas presenting lateral extensions or that are purely intraventricular, whereas the use of EEA was recommended for purely intrasellar craniopharyngiomas (9).

The approach chosen is determined based on the tumor’s location, pathology, consistency, and proximity to the pituitary stalk and optic chiasm; involvement of the third ventricle; history of prior surgeries; and the surgeon’s inclination based on experience and feasibility. Although both approaches are expected to remain feasible options for the treatment of craniopharyngiomas based on presentation, a growing number of case-series reports have provided evidence indicating specific surgical complications that are unique to each approach. Although previous meta-analyses have analyzed each approach (10, 11), comparative studies between TCA and EEA on this topic are rare. Comparative studies provide precise clinical descriptions that can be compared in a meta-analysis. To our knowledge, no meta-analysis has dealt with cranipharyngioma outcomes. Thus, the goal of this systematic review and meta-analysis was to collect all currently accessible evidence, including solely comparative studies, and determine whether there are any differences in clinical outcomes between TCA and EEA used to treat craniopharyngiomas.

## 2 Methods

### 2.1 Reporting guidelines and protocol registration

The guidelines of the Meta-analysis of Observational Studies in Epidemiology (MOOSE) (12) and the Preferred Reporting Items for Systematic Reviews and Meta-analysis guidelines were used in our investigation (13). The protocol was registered at http://www.crd.york.ac.uk/PROSPERO/ (CRD42021234801).

We developed a question that was based on population, intervention, comparison, and outcome (PICO). We conducted a critical evaluation based on the literature search and compiled the qualifying studies; their results were subsequently analyzed in a meta-analysis. The PICO question was as follows: Do patients with craniopharyngioma (population) treated surgically by EEA (intervention) compared to those treated by TCA (comparator) differ in surgical outcomes (outcome)?

### 2.2 Search strategy

Two expert reviewers (M. Na and B. Jang) conducted a literature search on July 28, 2020. The search included the MEDLINE and EMBASE databases *via* the Ovid interface, as well as the Cochrane library with no language restriction. Additionally, we manually searched the references of qualified studies to identify relevant research on July 30, 2022.

The following Medical Subject Headings terms were used to search all comparative studies in all logical permutations: “craniopharyngioma,” and; “transcranial,” or “craniotomy,” and “endoscopic,” or “endonasal” (**Supplementary Table 1**). We incorporated all publications that described prospective or retrospective cohort studies that addressed our PICO question.

### 2.3 Study selection

All studies from the literature search were registered into a reference management software, Endnote X8 (Clarivate Analytics, Philadelphia, United States). Two reviewers (M. Na and B. Jang) separately selected the studies based on predefined selection criteria after checking the title, abstract, and type of each article.

The exclusion criteria were as follows: inappropriate control in comparative studies, < 15 patients in the study, irrelevant results, duplicate data, letters, comments, editorials, case reports, reviews, or meta-analyses, and animal studies. After comparing the title, authors, and year of publication of all studies, we eliminated duplicate articles. On disagreement between the two reviewers, a third reviewer (K. Choi) intervened, and disagreements were debated until a consensus was reached. The full text of eligible publications was obtained after ineligible abstracts were removed and subjected to rigorous screening using the same inclusion and exclusion criteria.

### 2.4 Data extraction

Two reviewers (M. Na and B. Jang) independently extracted the pertinent patient data from the included studies. Disagreements between the reviewers were discussed till a consensus was reached. The following variables were extracted: the first author’s name, country, year of publication, study design, inclusion period, number of patients, type of TCA, tumor size, preoperative symptoms, and operative outcomes (extent of resection [EOR], visual outcome, hormonal outcome, complication outcomes: endocrine disorders, cerebrospinal fluid [CSF] leak, and others).

### 2.5 Risk of bias in individual studies

The methodological quality of eight selected studies was examined separately by two reviewers (M. Na and B. Jang) who were blinded to the authorship and journal using the Risk of Bias Assessment Tool for Non-randomized Studies (14). Unresolved differences among reviewers were addressed through discussion or review by the third author.

### 2.6 Statistical analysis

For each relevant outcome, the mean difference and odds ratio (OR) were utilized as summary statistics. The results of interest were described as forest plots; the weighted mean difference or OR, 95% confidence interval (CI), and relative weightings were represented by the middle of the square, horizontal line, and relative size of the square, respectively. A random-effects model was utilized to estimate pooled outcome measures from individual data of included studies. I^2^ statistics were used to determine the proportion of discrepancies between studies, with values of 25%, 50%, and 75% deemed as low, moderate, and high, respectively (15).

We used Review Manager version 5.4.1 (Cochrane Collaboration, Oxford, UK) to perform the statistical analysis for both main and sub-group analyses, and a P-value < 0.05 was considered statistically significant. Additionally, meta-regression was performed to analyze the gross total resection (GTR) rate in endocrinologic complication trends using web-r (http://www.web-r.org).

## 3 Results

### 3.1 Study selection and characteristics of included studies

**Figure 1:**
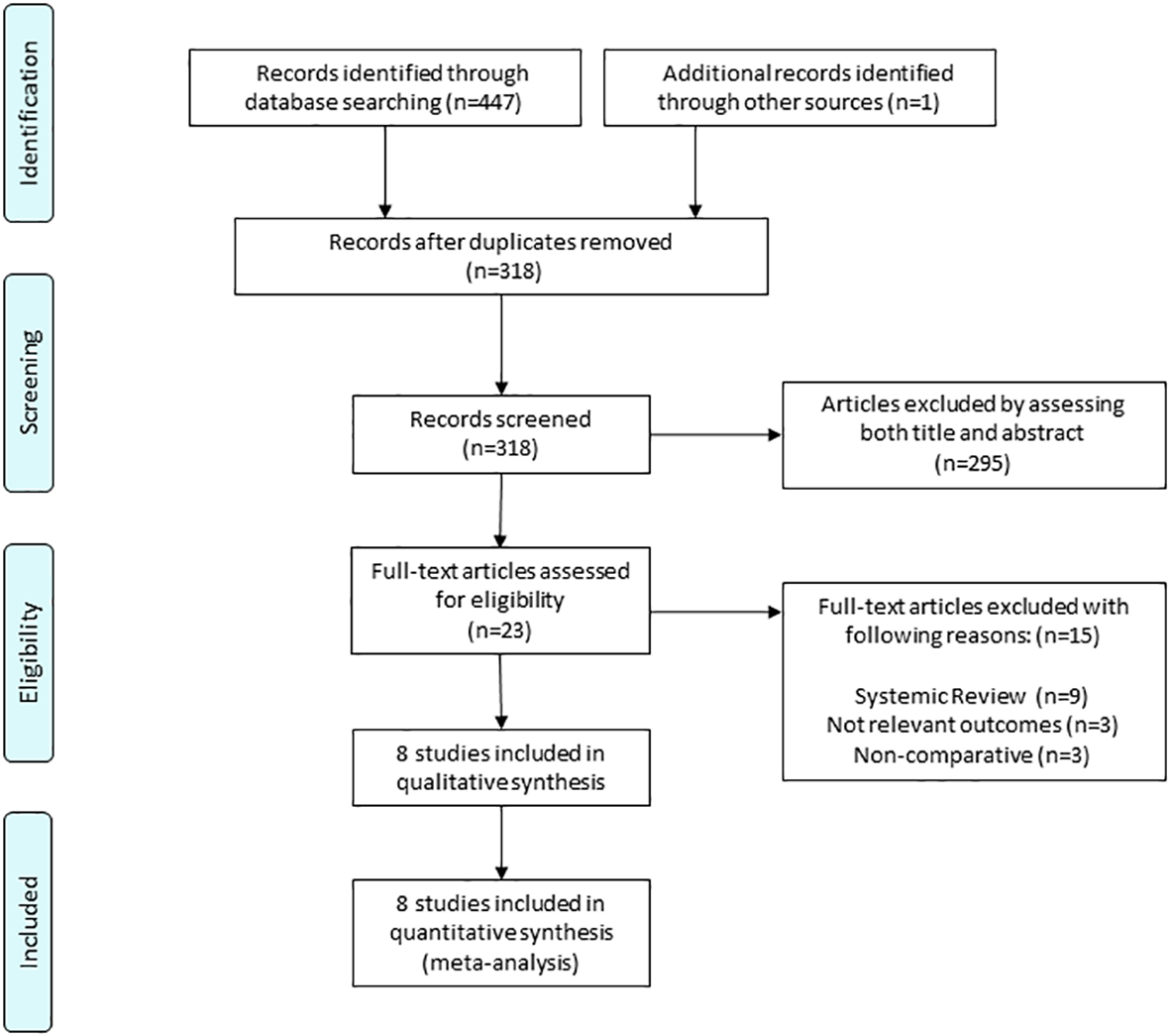
Flow diagram of identification of relevant studies.

Our literature search yielded eight eligible studies. On scanning the database, 447 records were found, and an additional study was identified from another source (**Figure 1**); 318 studies were assessed for eligibility after 130 duplicates were removed. Following this, 295 studies were eliminated after evaluating both titles and abstracts because of irrelevance to our study, leaving 23 potentially relevant studies. The full-text articles of these 23 studies were then obtained. We excluded 15 studies including systematic reviews (n = 9), those with irrelevant outcomes (n = 3), and non-comparative studies (n = 3), leaving eight studies (376 patients) to be included in the final meta-analysis (3, 4, 16–21).

**Figure 2:**
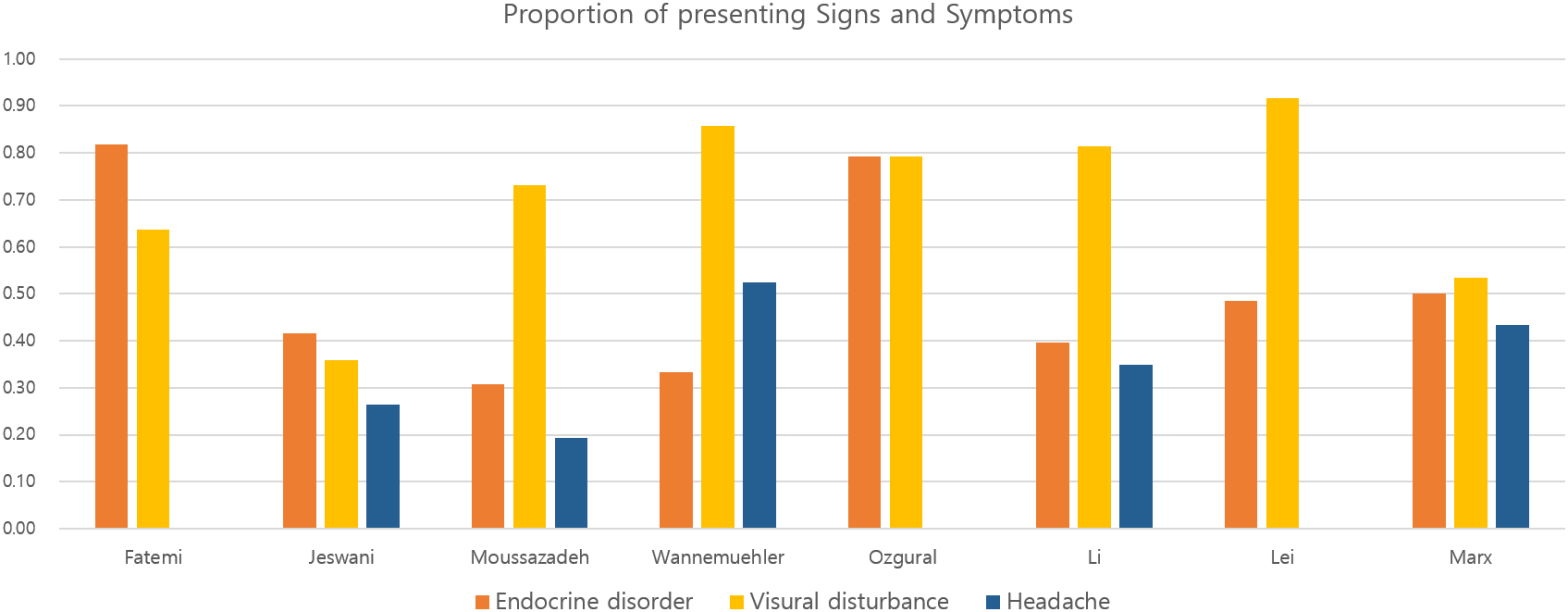
Bar plots representing the proportion of signs and symptoms in patients with craniopharyngioma in the included studies.

The eight retrospective observational studies (OS) were published between 2008 and 2020 with an enrollment period that ranged from 2000–2019 (**Table 1**). All tumor resections were performed in a single institution in all studies. When reported, the overall cohort’s mean age was 43.0 years, with a higher proportion of women (52%). The TCA group included a variety of methods, including pterional, orbitozygomatic, supraorbital, subfrontal, and transcallosal approaches. Although the descriptions in each article varied, intrasellar and significant laterally extended lesions were excluded, and tumors amenable to both approaches were included (**Table 1**). Therefore, we excluded 25 intrasellar tumors in one study (4) to reduce differences in inclusion criteria between the studies. Finally, the total number of patients was 401 and 376 in the systematic review and meta-analysis, respectively. Among 376 patients, 212 (56%) and 164 (44%) were surgically resected using EEA and TCA, respectively (**Table 2**). The most frequent presenting symptoms were visual disturbance (78%), hypopituitarism (48%), and headache (33%) (**Figure 2**).

**Table 1:** Characteristics of studies included in the systemic review.

Author, Year	Country	Study design	Study period	Number of patients	Male	Age (years)	Mean follow-up (months)	Transcranial type	Tumor location (Inclusion/Exclusion)
Fatemi, 2008 (18)	USA	R, 1 institution 1 surgeon	2000 - 2008	22	12	43.6	23.7	Supraorbital	Excluded parasellar lesions deemed unresectable by the endonasal route	
Jeswani, 2016 (17)	USA	R, 1 institution NR	2000 - 2013	53	28	45.2	34.6	Bifrontal Pterional OZ transcallosal	Included midline suprasellar/third ventricular lesions Excluded intrasellar lesions	
Moussazadeh, 2016 (3)	USA	R, 1 institution surgeons	2000 - 2015	26	7	50.8	35.2	Pterional	Included suprasellar lesions whose lateral extent does not pass the carotid bifurcation	
Wannemuehler, 2016 (16)	USA	R, 1 institution NR	2005 - 2015	21	13	50.1	10.5	Pterional OZ Bifrontal Transcallosal	Included tumors that were amenable to both approaches confirmed by another surgeon Excluded significant lateral extension	
Ozgural, 2018 (15)	Austria	R, 1 institution surgeons	2013 - 2017	24	15	32.3	NR	Pterional OZ	Excluded intrasellar lesions	
Li, 2018 (19)	China	R, 1 institution 4 surgeons	2011 - 2015	43	23	41.6	8.8	Pterional Supraorbital Subfrontal	Included only if the neurosurgeon confirmed that the tumor was amenable to both EEA and TCA	
Marx, 2020 (20)	Germany	R, 1 institution 1 surgeon	2001 - 2018	30	14	41	90.6	Pterional Supraorbital Transcallosal	Excluded intrasellar lesions	
*Lei, 2020 (4)	China	R, 1 institution NR	2013 – 2019	182	82	42.3	33	Pterion Subfrontal	*We excluded intrasellar lesions	

R, retrospective; EEA, endoscopic endonasal approach; TCA, transcranial approach; OZ, orbitozygomatic; NR, not reported.

*We excluded 25 intrasellar type craniopharyngiomas in quantitative synthesis (meta-analysis) which were resected by EEA alone to reduce differences from inclusion criteria in other studies.

**Table 2:** Tumor size, pathology, and extent of resection in patients with craniopharyngioma.

.	Approach	Number of patients	Size		Extent of resection			Pathology		
			Volume, cm^3^ mean (SD)	Length, mm mean (SD)	GTR, n (%)	NTR, n (%)	STR, n (%)	Adamantinomatous, n (%)	Papillary, n (%)	Mixed, n (%)
Fatemi, 2008 (18)	TCA	4	–	32 (14)	0	2	2	–	–	–
	EEA	18	–	31 (15)	3	9	6	–	–	–
Jeswani, 2016 (17)	TCA	34	9.5 (11.6)	–	–	–	–	23	9	2
	EEA	19	9 (9.8)	–	–	–	–	11	5	3
Moussazadeh, 2016 (3)	TCA	5	13.9 (7.8)	–	2	3	0	3	0	2
	EEA	21	8.5 (5.9)	–	19	2	0	7	3	11
Wannemuehler, 2016 (16)	TCA	12	7.8 (5)	–	7	0	5	11	1	0
	EEA	9	4.6 (4.7)	–	5	0	4	6	2	1
Ozgural, 2018 (15)	TCA	13	37.9 (22.4)		4	1	8	–	–	–
	EEA	11	24.6 (17.9)		9	0	2	–	–	–
Li, 2018 (19)	TCA	26	–	29.5 (9.5)	17	0	9	11	11	4
	EEA	17	–	25.2 (8.3)	11	0	6	6	8	3
Marx, 2020 (20)	TCA	13		–	43	11	3	–	–	–
	EEA	17	–	–	92	7	1	–	–	–
*Lei, 2020 (4)	TCA	57	–	–	7	0	6	–	–	–
	EEA	100	–	–	10	0	7	–	–	–

GTR, gross total resection; NTR, near total resection; STR, sub-total resection; TCA, transcranial approach; EEA, endoscopic endonasal approach; SD, standard deviation.

### 3.2 Extent of resection

**Figure 3:**
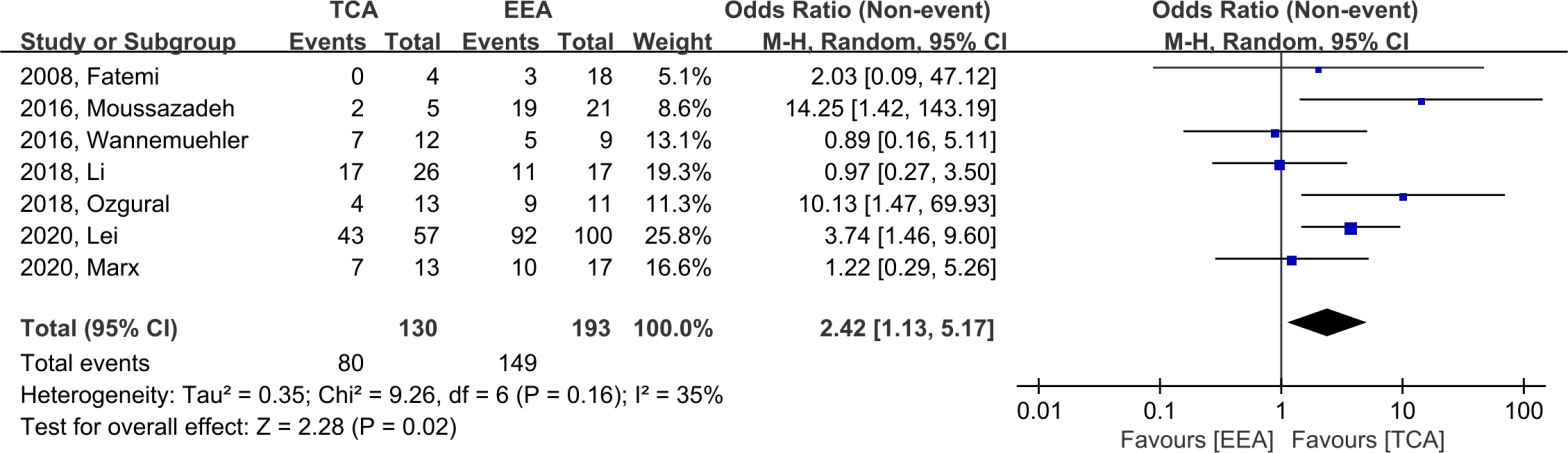
Forest plot comparing odd ratios (ORs) of extent of resection following TCA vs. EEA in craniopharyngioma patients. TCA, transcranial approach; EEA, endoscopic endonasal approach; CI, confidence interval; SD, standard deviation.

EOR was assessed in seven studies. We defined GTR as an event, and EEA demonstrated significantly higher likelihood of GTR (OR, 2.42; 95% CI, 1.13–5.17; p = 0.02; I^2^ = 35%). The incidence of GTR was 80/130 (61.5%) and 149/193 (77.2%) in TCA and EEA, respectively (**Figure 3**).

### 3.3 Visual outcomes

**Figure 4:**
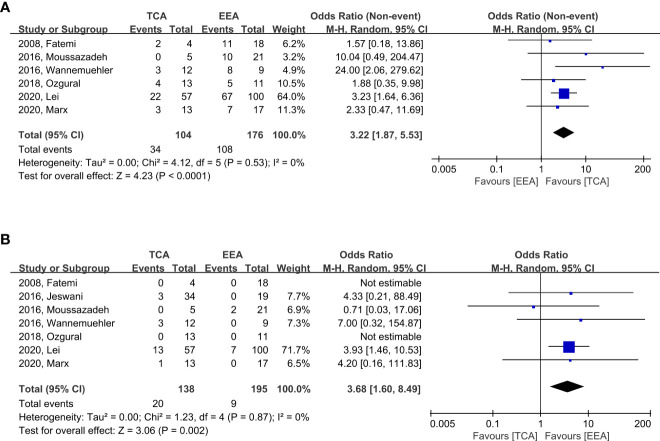
Forest plots comparing odd ratios (ORs) of visual outcomes following TCA vs. EEA in craniopharyngioma patients. **(A)** Visual improvement and **(B)** Visual deterioration. TCA, transcranial approach; EEA, endoscopic endonasal approach; CI, confidence interval; SD, standard deviation.

When compared to TCA, EEA demonstrated significantly higher likelihood of visual improvement (OR, 3.22; CI, 1.87–5.53; p < 0.0001; I^2^ = 0%; six studies); the incidence of visual improvement was 34/104 (32.7%) and 108/178 (60.7%) in TCA and EEA, respectively (**Figure 4A**). When utilizing TCA compared to EEA, there was a significantly higher likelihood of visual deterioration (OR, 3.68; CI, 1.60–8.49; p = 0.002; I^2^ = 0%; seven studies), with an incidence of 20/138 (14.5%) and 9/195 (4.6%) in TCA and EEA, respectively (**Figure 4B**).

### 3.4 Surgical complications

#### 3.4.1 Endocrine disorders

**Figure 5:**
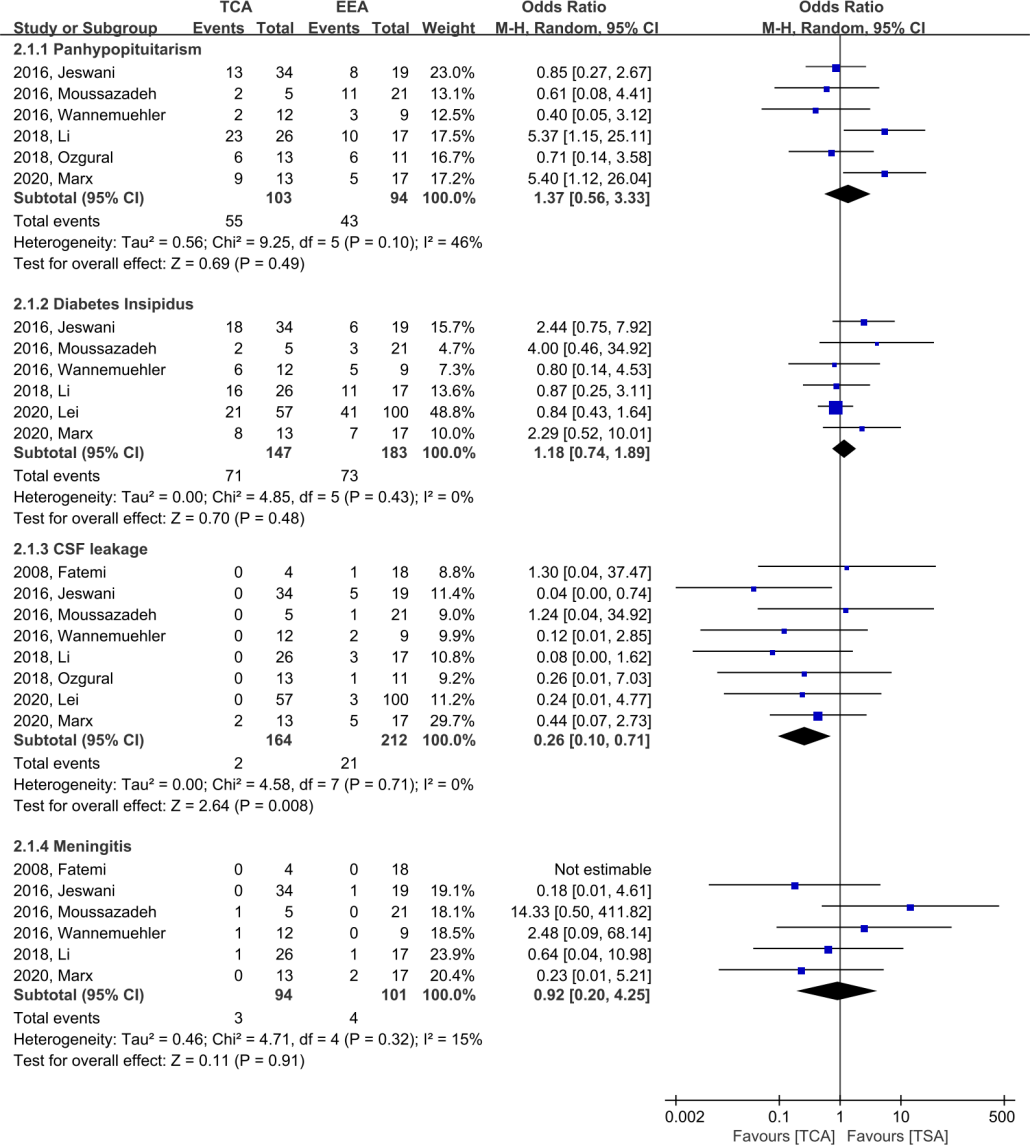
Forest plots comparing odd ratios (ORs) of complications following TCA vs. EEA in craniopharyngioma patients. TCA, transcranial approach; EEA, endoscopic endonasal approach; CI, confidence interval; SD, standard deviation; CSF, cerebrospinal fluid.

There was no significant difference between TCA and EEA with respect to panhypopituitarism (OR, 1.37; 95% CI, 0.56–3.33; p = 0.49; I^2^ = 46%; six studies), with an incidence of 55/103 (53.4%) and 43/94 (45.7%), respectively. In terms of diabetes insipidus (DI), there was no significant difference between TCA and EEA (OR, 1.18; 95% CI, 0.74–1.89; p = 0.48; I^2^ = 0; six studies), with an incidence of 71/147 (48.3%) and 73/183 (39.9%), respectively (**Figure 5**).

#### 3.4.2 CSF leakage and meningitis

When compared to EEA, TCA demonstrated a significantly lower likelihood of CSF leakage (OR, 0.26; 95% CI, 0.10–0.71; p = 0.008; I^2^ = 0%; eight studies), with an incidence of 2/164 (1.2%) and 21/212 (9.9%) in TCA and EEA, respectively (**Figure 5**). When EEA patients were divided into two groups according to the start date of the study period, the CSF leakage rate was reduced from 16.7% (14/84, five studies) before 2010 to 5.5% (7/128, three studies) after 2010 (**Supplementary Figure 1**). In terms of meningitis, there was no significant difference between TCA and EEA (OR, 0.92; 95% CI, 0.20–4.25; p = 0.91; I^2^ = 15%; six studies), with an incidence of 3/94 (3.2%) and 4/101 (4.0%), respectively (**Figure 5**).

### 3.5 Meta-regression analysis: Relationship between GTR and occurrence of endocrine disorders

**Figure 6:**
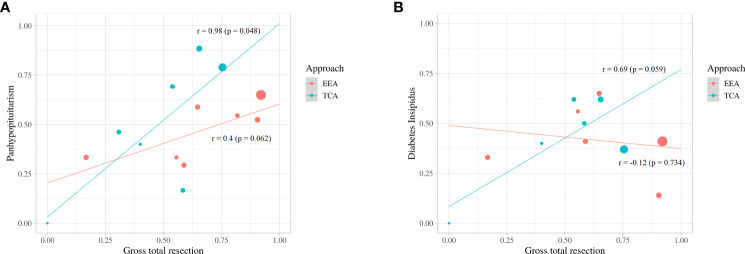
Scatter plot and linear-regression analysis between gross total resection and panhypopituitarism **(A)** and diabetes insipidus **(B)**.

Compared to EEA, TCA showed higher linear association between GTR and occurrence of panhypopituitarism (slope, 0.98; p = 0.048 vs. slope, 0.4; p=0.062) (**Figure 6A**). There was a linear association between GTR and occurrence of DI in TCA (slope, 0.69; p = 0.059), whereas an inverse association between GTR and occurrence of DI in EEA (slope, -0.12; p = 0.734) was observed (**Figure 6B**)

### 3.6 Risk of bias of the included studies

Using the Risk of Bias Assessment Tool for Non-randomized Studies system, the eight OS showed a low risk of bias in intervention measurement and blinding of outcome assessment and a high risk of bias in the selection of participants and confounding variables (**Supplementary Figure S2A**). Incomplete outcome data and selective outcome reporting were high risks of bias in two (20, 21) and four studies (**Supplementary Figure S2B**) (4, 16, 20, 21), respectively.

## 4 Discussion

This systematic review and meta-analysis examined surgical outcomes of craniopharyngiomas treated with EEA and TCA. To the best of our knowledge, this is the first meta-analysis providing direct comparison based on comparative studies. We found that compared to TCA, EEA showed favorable EOR and visual outcomes. EEA also showed less likelihood of endocrine disorders, although this was not statistically significant. Compared to EEA, TCA showed less likelihood of CSF leakage, while the occurrence of meningitis was not significantly different between the approaches. These results suggest that when both approaches are feasible, EEA has favorable surgical outcomes.

Currently, the optimal management for treating patients with craniopharyngioma is controversial. GTR of craniopharyngioma was formerly considered to be challenging due to perioperative complications; therefore, sub-total resection (STR) followed by adjuvant radiotherapy was deemed as an alternative treatment option (7, 22). Although STR followed by adjuvant radiotherapy and GTR had comparable disease control rates, long-term complications after radiotherapy, such as hypopituitarism and cognitive impairment, have emerged (23). As a result, surgery remains the mainstay of treatment and offers radical resection, which maximizes the possibility of oncological cure (6, 8, 24, 25). The ability to accomplish GTR is an important factor in deciding surgical approaches. Liu et al. (6) emphasized the importance of a tailored approach for individual patients depending on the extent of the tumor and its proximity to neighboring structures in determining the optimal treatment strategy. TCA provides direct access to the parasellar compartments and is useful for tumors that extend laterally beyond the internal carotid artery bifurcation (3). However, EEA provides direct access to the anterior skull base and is appropriate for intrasellar lesions (26). In this study, EEA resulted in a significantly higher likelihood of GTR in lesions where both approaches are viable (77.2% vs 61.5%; OR, 2.24; p = 0.02). EEA allows for direct visualization and dissection of tumors and adhesive neurovascular structures, increasing the likelihood of complete resection.

Endocrine dysfunction adversely affects health-related quality of life and seems inevitable after surgery (27–29). The pituitary stalk connects the pituitary gland to the hypothalamus and maintains the hypothalamic-pituitary function (2). The relationship between the tumor and stalk is critical for postoperative endocrine dysfunction, and the Kassam classification focused on this relationship (30). Dho et al. (2) reported that trans- and retro-infundibular tumors were associated more with endocrinological deterioration than pre-infundibular tumors according to the Kassam classification, and centrally located tumors were significantly associated with endocrinological deterioration than peripherally located tumors. A previous meta-analysis found that patients treated with GTR had a considerably higher incidence of panhypopituitarism and DI than those treated with STR (27). In this study, there was no significant difference in the incidence of panhypopituitarism and DI between TCA and EEA. In a linear regression, the incidence of panhypopituitarism and DI increased significantly with increasing GTR ratio in TCA, whereas the incidence of panhypopituitarism increased slightly and DI showed a tendency to decrease with increasing GTR ratio in EEA. Compared to TCA, EEA allows for a more direct view of the skull base, allowing for early identification of the pituitary stalk and GTR while preserving the stalk. Chen et al. (31) reported that when craniopharyngiomas were resected *via* EEA, stalk preservation significantly lowered endocrine dysfunction without decreasing the rate of GTR and without increasing the rate of tumor recurrence.

We found that EEA resulted in a significantly higher likelihood of visual improvement when compared to TCA (60.7% vs. 32.7%, p < 0.0001), whereas TCA resulted in a significantly higher likelihood of visual deterioration when compared to EEA (14.5% vs. 4.6%, p = 0.002), and the results were comparable to those reported in a previous meta-analysis (11). These results support the evidence that EEA has an advantage over TCA by increasing visual improvement but reducing visual deterioration. Stefko et al. also demonstrated that EEA improves the visual field as well as visual accuracy (32). This is because EEA allows for early decompression of the optic apparatus without retraction and superior visualization of superior hypophyseal arteries originating from the internal carotid artery.

CSF leakage was shown to be statistically more prevalent in EEA compared to TCA (9.9% vs. 1.2%, p = 0.008). When EEA was originally introduced, the increased possibility of postoperative CSF leakage was a major complication. To access the tumors, EEA penetrates through the nasal cavity and deconstructs the anterior skull base due to the pathway of the approach. However, with the introduction of skull base reconstruction techniques using a pedicled vascularized nasoseptal flap, first introduced in 2006, this risk has been considerably decreased to approximately 5% (33, 34). In our study, it was confirmed that the CSF leakage rate was as low as 5.5% in the studies with a study period after 2010 (33, 34). The development of multi-layer skull base reconstruction techniques, including gasket-seal, artificial collagen dura mater, and artificial bone substitute, and increased surgeon experience are expected to further reduce the rate of CSF leakage.

### 4.1 Strengths and limitations

This meta-analysis adhered strictly to its selection criteria and the Preferred Reporting Items for Systematic Reviews and Meta-analysis guidelines. This study has several strengths. First, although direct comparative studies of craniopharyngioma resection using TCA vs. EEA are uncommon, we only incorporated this type of research to avoid intra-study variability which affects indirect comparisons, improve the validity of the results, and provide summary statistics. Second, we could reduce selection bias because most of the studies attempted to include tumors that were amenable to both approaches. Lei et al. (4) reported four types based on the location of the tumors, and we excluded the intrasellar type to avoid violating the inclusion criteria of other studies.

However, our study has some limitations. First, all included studies were retrospective in nature. Second, two of the eight studies reported incomplete outcome data and had selective outcome reporting, such as tumor size, pathology, and adjuvant radiotherapy. (**Supplementary Figure 2B**). However, missing data were not analyzed in this study and did not significantly impede the conclusions. Third, we were unable to analyze other complications such as hydrocephalus, nerve injury, cerebral infarction, cognitive dysfunction, and hemorrhage, as only a few studies have reported these parameters for their patients. Therefore, it is important to carefully interpret the results of this study, and a further well-designed study is warranted.

### 4.2 Conclusions

We found that when both approaches can completely resect the tumor, EEA outperforms TCA in terms of GTR rate and visual outcomes, as well as favorable results in terms of complications other than CSF leakage, such as panhypopituitarism and DI, considering the meta-regression results. Although knowledge of and competence in traditional microsurgery and endoscopic surgery are essential in surgical decision-making for craniopharyngioma treatment, when both approaches are viable, EEA is associated with favorable surgical outcomes.

## Data availability statement

The original contributions presented in the study are included in the article/**Supplementary Material**. Further inquiries can be directed to the corresponding author.

## Author contributions

K-SC contributed to the conceptualization, supervision, and project administration. MN, BJ, TL, WK, and YC contributed to the methodology, formal analysis, data curation, and writing the original draft. H-GS, CA, JK, JL, SK, and HL contributed to the validation, writing, review, and editing. All authors contributed to the article and approved the submitted version.

## Funding

This work was supported by the Bio & Medical Technology Development Program of the National Research Foundation (NRF) funded by the Korean government (MSIT) (NRF-2019M3E5D1A01069356) and the National Research Foundation of Korea (NRF) grant funded by the Korea government (MSIT) (NRF-2022R1A5A1022977) to K-SC.

## Conflict of interest

The authors declare that the research was conducted in the absence of any commercial or financial relationships that could be construed as a potential conflict of interest.

## Publisher’s note

All claims expressed in this article are solely those of the authors and do not necessarily represent those of their affiliated organizations, or those of the publisher, the editors and the reviewers. Any product that may be evaluated in this article, or claim that may be made by its manufacturer, is not guaranteed or endorsed by the publisher.

## Abbreviations

CSF, Cerebrospinal fluid; DI, Diabetes insipidus; EEA, Endoscopic endonasal approach; EOR, Extent of resection; GTR, Gross total resection; OR, Odds ratio; OS, Observational studies; PICO, Population, intervention, comparison, and outcome; RE, Random-effects; STR, Sub-total resection; TCA, Transcranial approach.
