# Supplementary material for: Craniopharyngioma resection by endoscopic endonasal approach versus transcranial approach: A systematic review and meta-analysis of comparative studies
Source: Front Oncol. 2022 Nov 30;12:1058329. doi: 10.3389/fonc.2022.1058329 (PMC9748146; doi:10.3389/fonc.2022.1058329)

**Supplementary Table 1.** Comprehensive list presenting the search strategy.

EMBASE & MEDLINE

| 1 | Craniopharyngioma.mp. [mp=tx, bt, ti, ab, ct, sh, hw, kw, sa, nm, fx, kf, ox, px, rx, an, ui, ds, on, sy] | 8940 |
| --- | --- | --- |
| 2 | Transcranial.mp. [mp=tx, bt, ti, ab, ct, sh, hw, kw, sa, nm, fx, kf, ox, px, rx, an, ui, ds, on, sy] | 96190 |
| 3 | Craniotomy.mp. [mp=tx, bt, ti, ab, ct, sh, hw, kw, sa, nm, fx, kf, ox, px, rx, an, ui, ds, on, sy] | 56747 |
| 4 | Endoscopic.mp. [mp=tx, bt, ti, ab, ct, sh, hw, kw, sa, nm, fx, kf, ox, px, rx, an, ui, ds, on, sy] | 391540 |
| 5 | Endonasal.mp. [mp=tx, bt, ti, ab, ct, sh, hw, kw, sa, nm, fx, kf, ox, px, rx, an, ui, ds, on, sy] | 11479 |
| 6 | 2 or 3 | 149747 |
| 7 | 4 or 5 | 394869 |
| 8 | 1 and 6 and 7 | 447 |

**Supplementary Figure 1.** Forest plots comparing odd ratios (ORs) of CSF leakage following TCA versus EEA in craniopharyngioma patients before and after 2010.


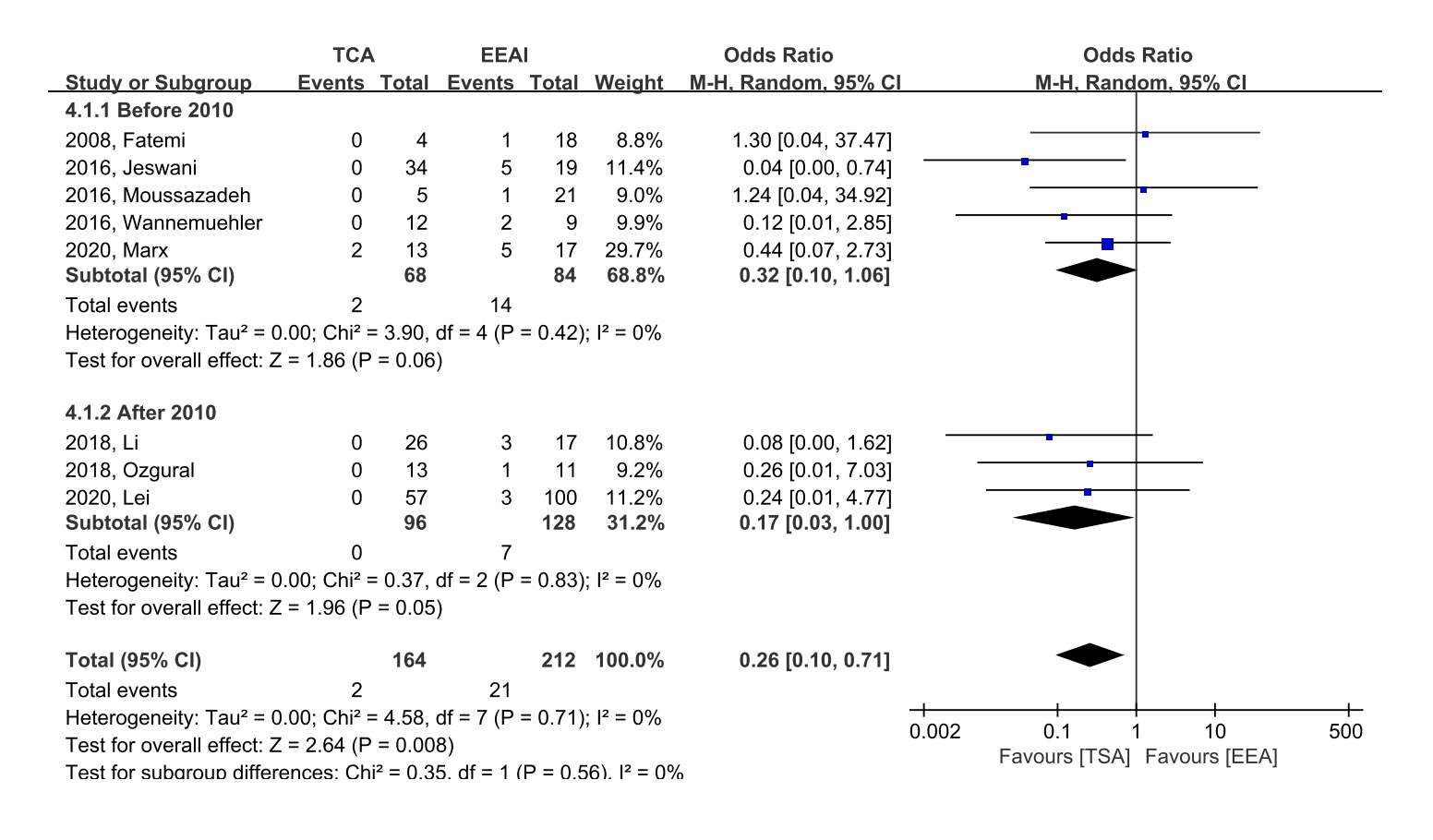


**Supplementary Figure 2.** Quality of the included studies


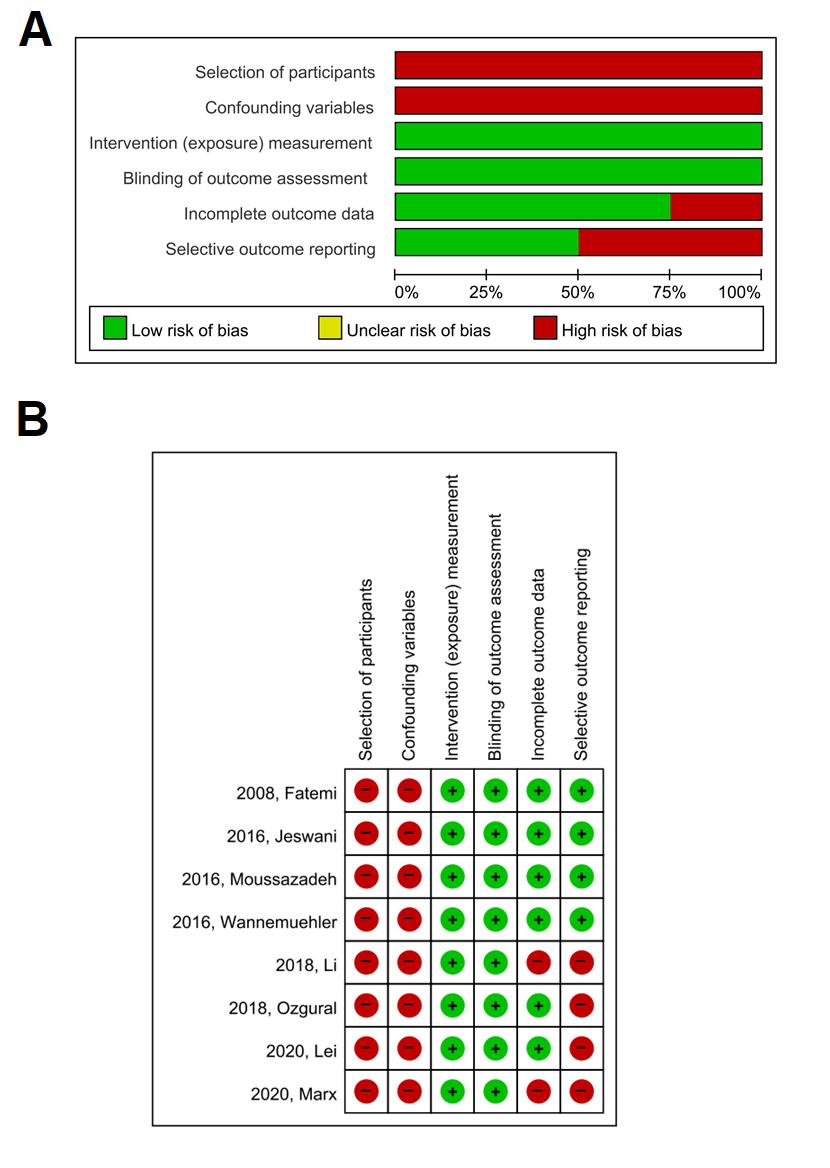

Supplement: Supplementary file 1 [file DataSheet_1.docx]
